# The Italian Registry of GH Treatment: electronic Clinical Report Form (e-CRF) and web-based platform for the national database of GH prescriptions

**DOI:** 10.1007/s40618-018-0980-3

**Published:** 2018-11-15

**Authors:** F. Pricci, M. Villa, F. Maccari, E. Agazio, D. Rotondi, P. Panei, P. Roazzi

**Affiliations:** 1 Department of Cardiovascular, Endocrine-Metabolic Diseases and Aging, Istituto Superiore di Sanità, Rome, Italy; 2 Information Technology Service, Istituto Superiore di Sanità, Rome, Italy; 3 Grant Office and Technology Transfer, Istituto Superiore di Sanità, Rome, Italy; 4 Health Technology Assessment, Istituto Superiore di Sanità, Rome, Italy

**Keywords:** Growth hormone, Pharmacoepidemiology, Epidemiological monitoring, Case reports, Registry, Database

## Abstract

**Background:**

In Italy, the utilization and the reimbursement of Growth Hormone (rGH) therapy by the National Health System (Servizio Sanitario Nazionale) are regulated by the “Note #39” included in the “Notes for the use of drugs” by the Italian Medicines Agency (AIFA), which are published in the Official Gazette, thus having the force of law. The “Note #39” establishes the diagnosis for which the reimbursement is granted and confirms the assignment of the national health surveillance on the use of GH therapy to the Italian National Institute of Health, requesting its computerization.

**Aim:**

The aim of this work was to realize a dedicated electronic Clinical Report Form based on the mandatory data requested by the Note #39 and allowing the online reporting of the rGH prescriptions by the regional accredited centers.

**Results and Conclusions:**

This interface is at the base of the national database of the Italian Registry of GH Treatment, which allows obtaining and managing correct and complete data to provide public health surveillance on GH therapy, both at national and local levels, necessary for policymakers decisions. In addition, this national database could be a useful instrument for improving knowledge about aspects of this treatment still under discussion.

## Introduction

Recombinant growth hormone (rGH) has been used since 1985 and is applied both in Growth Hormone (GH)-deficiency and in non-GH-deficiency diseases, such as Turner (TS) and Prader Willi (PWS) syndromes, chronic renal failure, children born Short for Gestational Age (SGA) and Idiopathic Short Stature (in the USA, but not in Europe so far) in pediatric age. Its use is foreseen also in adulthood in cases of GH deficit due to hypopituitarism or genetic causes.

Thus, rGH treatment is a chronic therapy, with the peculiarity of being performed mostly in the particularly sensitive period of childhood and for a long time. Nevertheless, rGH has not been sufficiently analyzed in terms of safety, efficacy and effectiveness by large clinical studies, probably because of lacking exhaustive databases and for the heterogeneity of disease phenotypes. Moreover, the starting age of patients and duration of treatment are highly variable, depending on the pathology and the therapy goals.

Only a few databases are available on rGH treatment. Most of them are GH registries created by pharmaceutical companies, with the main aim of evaluating efficacy and safety. A recent review reported a combined analysis of large-scale GH registries sponsored by pharmaceutical companies, concluding that published data supported an increased risk in second malignancies, but not in mortality or malignancy in children or adults treated with rGH. Furthermore, type 2 diabetes risk may be increased in GH-treated children and adults, even though this appears to be confined to those with classical risk factors, including age and Body Mass Index [1].

A meta-analysis including no-profit studies, such as those conducted on Dutch and French national GH registries, showed a significant increase in all-cause mortality but no significant increase in cancer and cardiovascular mortality. The cancer incidence and the risk for second neoplasms were significantly increased, highlighting the biases that should be considered, such as heterogeneity of population, different age (adult or pediatric cohorts), different diagnosis, limited sample size and low event rate [2].

The European project “SAGhE (Safety and Appropriateness of Growth Hormone Treatments in Europe) included 24,232 patients treated in childhood with rGH in 8 European countries [3]. Data from the French study evidenced that mortality rates were increased in the population of adults treated as children with recombinant GH, particularly in those who had received the highest doses. Specific effects were detected in terms of death due to bone tumors or cerebral hemorrhage [4]. This warning was not confirmed by data from Belgium, The Netherlands, and Sweden, where a similar distribution of causes of death was not observed [5]. General results from SAGhE do not support a carcinogenic effect of rGH, but highlight an unexplained trend in cancer mortality risk in relation to GH dose in patients with previous cancer, and the indication of possible higher risk for bone and bladder cancers and Hodgkin’s lymphoma [6].

As for other reports, confounding aspects were the different GH- and non-GH-deficiency diseases, the inherent risks for mortality and malignancy for other pathologic conditions, and the different replacement therapies with GH or the supra-physiological supplementation of GH.

To sum up, long-term safety of rGH therapy is still under investigation, needing appropriate and detailed studies.

In addition to safety, a relevant issue is represented by the cost of this therapy, which leads to control on its utilization, as performed in The Netherlands [7] or Italy. A further problem related to rGH is represented by its illegal use as a doping drug for its anabolic and lipolytic activity and its difficult detection [8].

In Italy, a special system of pharmacovigilance for medicinal products presenting high risks or costs has been in force since 1993 (Ministerial Decree, November 29, 1993) and a national registry specifically dedicated to rGH therapy, the National Registry of Growth Hormone therapy (Registro Nazionale degli Assuntori dell’Ormone della Crescita-RNAOC), has been established at the National Institute of Health (ISS). This registry has been collecting the national rGH prescriptions since the late 1980s, assembling data from paper communications derived from university and hospital centers.

In 2004, in the “Notes for the use of drugs”, the Italian Medicines Agency (AIFA) established criteria for the reimbursement of medicines by the Italian National Health System (Servizio Sanitario Nazionale—SSN) and with the Note #39 defined the diagnoses for which rGH treatment could be refunded.

These “Notes”, and their subsequent updates, are published in the Italian Official Journal [9] and have force of law. In its first version, the Note #39 confirmed that ISS was in charge of conducting the national epidemiological surveillance on rGH prescriptions and in 2007 it required the computerization of this activity [10]. Considering the Italian regionalized health system, the Note #39 required also the establishment of regional commission dedicated to supervise GH therapy in terms of controls and authorizations [9–11].

The aim of this work was to design a dedicated electronic Clinical Report Form (e-CRF) to obtain a national database on this treatment as foreseen by the Note #39, thus including the requested data and the permitted diagnosis and criteria, to realize a computerized RNAOC, useful to perform the national pharmacosurveillance on rGH therapy through the collection of the medical prescriptions.

## Methods

### Dataset

The dataset for collecting adequate information about the prescriptions was planned on the basis of the warnings by AIFA and of scientific literature (relevant clinical trials or international guidelines) and adapted in an electronic format.

### e-CRF and web platform

The e-CRF was structured in records, containing the patient clinical data.

The electronic form makes use of controls about correctness of the input and multiple choices for exactly defining specific information. Check boxes, drop-down lists, calendar for dates were included with the aim of reducing free text as much as possible.

A dedicated web-based platform was set up to enable data entry and collection through the e-CRF. This system allows information to be received, authenticated, tracked, and stored. The platform provides a connection based on username and password, automatic disconnection, secure communication (HTTPS), traceability of changes (date/time, user) and of actions (connection, disabling). Data are stored in a relational database. Backup is performed twice a day.

### Structure/roles/accreditation

The architecture of the system took account of the different assignments of the entitled parties. The roles of “administrator”, “auditor”, “supervisor” and “users” were planned, and permissions and conditional displays were based on these roles.

### Procedures/process

Only the authorized users could fill the e-CRF and the warning of a prescription of therapy is declared by the “submission” of a completed visit. Quality checks are present at different levels. During the data entry phase, real-time checks are performed: automatic verification of missing data, format of input data (date format, num vs alpha); data out of bounds, interval. When saving data, consistency checks and compatibility rules are performed. Finally, backoffice controls were included as statistical checks to highlight significant differences with respect to expected values.

### Helpdesk

Helpdesk activity was planned to support clinical units, physicians and regional authorities through a point of contact for users to gain assistance in troubleshooting, get answers to questions, and solve problems. The expected main issues should be related to guidance about the electronic submission, errors in logs entered and updating of accreditations to the system, both for users and clinical centers.

### Paper notifications

The rGH therapy notifications, sent to ISS as paper notice before the computerization of RNAOC, were entered in a dedicated database and analyzed for treated subjects, follow-up visits and diagnoses.

## Results

A national expert panel, with specialists from ISS and members of scientific societies and institutions, was nominated and established the dataset for the form. The e-CRF was designed in accordance and the system became operational in 2011.

The RNAOC-dedicated e-CRF is available for free to the accredited users, via a simple Internet connection with an optimal level of availability and security, using widespread browsers.

The access takes place through a secure area of the RNAOC website.

The procedures of data collection from clinical units to the RNAOC are in agreement with the Italian law on privacy (D.Lgs 196/2003) and will be updated according to the incoming European regulation.

The architecture of the system is based on the clinical units where patients are followed up. They are identified by regions on the basis of defined criteria. The structures accredited to the RNAOC e-CRF are the Centers, such as hospitals/universities including one or more Operating Units (OU), and the Regions, consisting of the Regional Commission and the administrative figures.

The accreditation procedures entail the autonomous decision by the Regions about the adhesion to the electronic system.

To accomplish the accreditation, regions identify and communicate to the RNAOC the Centers/OUs that are authorized to prescribe rGH and the corresponding persons in charge as “supervisors”. The OU, associated with the corresponding Center, could be a clinical unit or a day hospital unit.

The system needs to take in account the roles of controller and of clinician, considering the different activities of central and local competent authorities and of the clinical centers.

The role of “auditor” corresponds to the local authority, region and/or “Regional Commission for GH”, as foreseen by the AIFA act, and to the general director of the hospital. The “auditor” has to monitor aspects of this therapy, such as costs, authorizations to treat or could oversee clinical unit activity.

The role for physicians is classified as “supervisor”, corresponding to the responsible for the clinical unit, or “user”, created and managed by the supervisor. Their task is the management of patients in terms of entering demographic and clinical data.

The “administrator” (ISS) manages accreditations both for clinical centers and for auditors and supervisors.

The access as “auditor”, “supervisor” and “user” is regulated by username and password; and the latter is automatically generated by the system and modifiable by the user. A section for managing roles and the personal profile is present.

The web e-CRF includes a series of sections containing the data necessary for the inclusion and follow-up of the patients, and the organization of the data in a database interfaced with statistical analysis programs.

For what concerns the form, the e-CRF contains the dataset defined by the experts group, which includes a minimum data set of mandatory data derived from the law and essential for the activities of pharmacosurveillance. It is unique for all the diagnoses, i.e., childhood and adults, and has been planned as a medical record to allow the input of useful information for the complete clinical management of the patient.

The form is divided in two parts containing the patient’s personal data (patient card) and the reports of the clinical information (visit cards), respectively.

To accomplish the privacy protection act, an information notice is available in the patient card to be provided by the physician to the patient, including the description of personal data treatment, in terms of objective, methods, ownership. It explains that consent to data processing cannot be refused because it is required by law.

From the operational point of view, the physician (supervisor and user), associated with an OU as patients as well, can enter a new patient or a new follow-up visit of a patient already registered in the system.

The visit is organized in sections and includes mandatory information, such as Residence, Diagnosis and Therapy. Additional sections, as Medical History, Physical Examination, Exams, Intercurrent Diseases and Adverse Events, and Suspension, are useful for pharmacosurveillance.

Residence information is necessary because the SSN, organized under the Ministry of Health, is administered on a Regional basis; thus, the reimbursement of medicine is linked to the Regional residence of the patient.

Diagnosis section is compliant to the Note #39 currently in force [12] and the allowed diagnoses are classified according to the age, in a drop-down list.

Moreover, a list of diagnoses from Note #39 could be selected in case of input of “historical” data, allowing managing the whole medical history of patients.

Therapy section is the core of the collection of data and includes mandatory fields as commercial name of medicines (as authorized by AIFA), dosage and schedule. A utility, consisting in a small automatic computation program, can calculate the dosage from dose (in mg/day or week) and weight, useful because of the difference in dose of rGH therapy in relation to the diagnosis. In addition, the user could select a previous therapy and simply confirm it or change it. This section can originate a document (pdf), which can be used for prescription.

Partial data entry is allowed, with the possibility of completing the form in a different moment.

At the end of the visit, if all the mandatory fields and records are completed, the user can “submit” it, thus implying the communication of the prescription of therapy to the RNAOC. At this point, the visit cannot be further modified and it is entered in the RNAOC database for pharmacosurveillance analysis.

Updating of the procedures is envisaged and applied to upgrade the e-CRF on the basis of changes in legal provision, as for the Note #39 published in 2014. A continuous updating is necessary for the accreditations of clinical centers and users. This activity is strictly dependent on regional decisions and a continuous collaboration between ISS and Regional Commissions representatives is crucial.

A periodic newsletter to all the users is scheduled, with a report on the main items on electronic system and update about rGH therapy and meetings.

### Preliminary results on the utilization of e-CRF

At the end of 2017, 19 Italian Regions + the 2 Autonomous Provinces (AP) have joined the e-CRF of the RNAOC and 151 clinical centers with 217 medical outpatients’ facilities have been indicated as officially recognized “prescribing centers”. The users were 14 “auditors”, 221 “supervisors” and 117 “users” (Table 1).

**Table 1:** Accreditations to RNAOC e-CRF, divided by regions or autonomous provinces (AP)

Region/AP	Centers (n.)	OUs (n.)	Auditors	Supervisors (n.)	Users (n.)
Abruzzo	6	8	Yes	7	5
Aosta Valley	1	1		1	2
Apulia	15	20	Yes	20	21
Basilicata	7	8		7	1
Bolzano	1	1		1	
Calabria	9	14	Yes	14	6
Campania	2	2		2	1
Emilia Romagna	17	25	Yes	26	14
Friuli Venezia Giulia	5	7	Yes	7	3
Lazio	11	20		24	11
Liguria	4	5	Yes	5	7
Lombardy	32	43	Yes	46	19
Marches	10	15	Yes	15	7
Molise	3	6	Yes	4	2
Piedmont	0	0		0	0
Sardinia	4	7	Yes	7	6
Sicily	9	10	Yes	10	6
Trento	1	1		1	
Tuscany	10	17	Yes	16	5
Umbria	4	7	Yes	8	0
Veneto	0	0	Yes	0	0
Total	151	217	14	221	117

It is noted that Piedmont, Campania, Lazio and Veneto are endowed with local databases for collecting rGH prescriptions and joined also RNAOC web system both for having changed the system in the course of time or on specific request by other Regions in case of patients coming from the latter ones.

Concerning the data, quality control of the database was performed on the whole database to verify missing or incorrect data that the system could not reveal. As an example, the declared age range must be consistent with the type of diagnosis and correct rGH dosage can be verified if weight is indicated. Afterwards, the Responsible of the medical outpatients’ facilities was contacted to verify and eventually correct the input.

Preliminary data on clinical information report that at the end of 2017, the analysis of notifications forwarded by the registered national specialist centers through RNAOC e-CRF showed a total of 5712 rGH-treated patients with the requested specific diagnoses, clinical markers and prescriptions, as reported by the AIFA regulation (Table 2).

**Table 2:** rGH-treated subjects and follow-up visits notificated through RNAOC e-CRF at December 31, 2017

Region/AP	Subjects (n.)	Follow-up visits (n.)
Abruzzo	669	2856
Aosta Valley	0	0
Apulia	1863	7779
Basilicata	60	136
Calabria	57	91
Campania	2	4
Emilia Romagna	559	1730
Friuli Venezia Giulia	86	308
Lazio	83	180
Liguria	178	243
Lombardy	286	948
Marches	735	3221
Molise	119	552
Piedmont	0	0
Sardinia	166	861
Sicily	554	1392
Trento/Bolzano	3	3
Tuscany	58	182
Umbria	234	1640
Veneto	0	0
Total	5712	22,126

The classification of patients under rGH treatment according to the diagnoses specified in the Note #39 demonstrated a prevalence of subject in childhood (80.25%) (Table 3). Doses of rGH reported by the clinicians according to the diagnosis are consistent with the doses suggested by scientific literature (Table 4).

**Table 3:** Subjects (absolute numbers and percentages) under rGH treatment, classified according to the diagnoses specified in the Note #39

Diagnosis as stated by Nota #39	Subjects			
	*N*°	%	*n*°	%
*First 2 years of life*	*25*	*0.44*		
Deceleration of growth rate or hypopituitarism and/or hypoglycemia			25	0.44
*Childhood*	*4584*	*80.25*		
GH deficiency (GHD)			3942	69.01
Turner syndrome (TS)			196	3.43
Chronic renal failure			28	0.49
Prader-Willi syndrome (PWS)			72	1.26
Short stature homeobox-containing gene (SHOX) deficiency			35	0.61
Small for gestational age (SGA)			311	5.44
*Transition-age*	*151*	*2.64*		
Genetic mutation			28	0.49
Panhypopituitarism/PWS			40	0.70
GH < 6 µg/L (insulin tolerance test)			16	0.28
GH < 19 µg/L (GH-realising hormone + arginine)			67	1.17
*Adulthood*	*834*	*14.60*		
Hypophysectomy			336	5.88
Hypopituitarism			497	8.70
Congenital GH deficiency			1	0.02
*Out of notice*	*102*	*1.79*		
First 2 years of life			1	0.02
Childhood			91	1.59
Transition-age			3	0.05
Adulthood			7	0.12
*No info*	*16*	*0.28*	16	0.28
**Total**	**5712**	**100**	5712	100

**Table 4:** Doses of rGH reported by the clinicians according to the diagnosis (Doses are both in mg/die and in mg/week. The medians have also been calculated)

Diagnosis as stated by Nota #39	Dose (mg/die)			Dose (mg/wk)		
	Median	5°	95°	Median	5°	95°
*First 2 years of life*						
Deceleration of growth rate or hypopituitarism and/or hypoglycemia	0.029	0.011	0.4	0.2	0.083	2.55
*Childhood*						
GH deficiency (GHD)	0.03	0.02	0.04	0.209	0.16	0.29
Turner syndrome (TS)	0.038	0.014	0.051	0.27	0.18	0.4
Chronic renal failure	0.035	0.021	0.05	0.213	0.175	5
Prader-Willi syndrome (PWS)	0.023	0.011	0.037	0.16	0.061	0.25
Short stature homeobox-containing gene (SHOX) deficiency	0.038	0.028	0.05	0.26	0.166	0.324
Small for gestational age (SGA)	0.033	0.024	0.048	0.24	0.145	0.346
*Transition-age*						
Genetic mutation	0.034	0.032	0.045	–	–	–
Panhypopituitarism/PWS	0.013	0.001	0.05	0.04	0.025	0.2
GH < 6 µg/L (insulin tolerance test)	0.012	0.005	0.022	0.045	0.007	0.15
GH < 19 µg/L (GH-realising hormone + arginine)	0.013	0.002	0.036	0.039	0.016	0.203
*Adulthood*						
Hypophysectomy	0.004	0.001	0.4	0.022	0.01	1.96
Hypopituitarism	0.005	0.001	0.4	0.047	0.009	1.61
Congenital GH deficiency	–	–	–	–	–	–

**Fig. 1:**
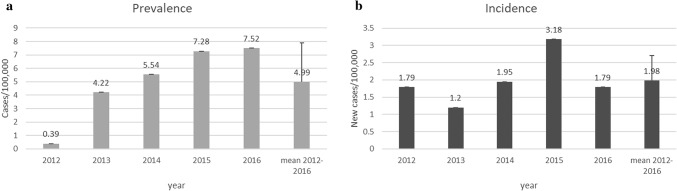
Prevalence (**a**) and incidence (**b**) of rGH treatment in Italy, according to the web-RNAOC database

Prevalence and incidence of rGH treatment for the years 2012–2016, which represent the years of operative e-CRF, indicate a mean of 4.99 ± 2.9 treated/100,000 and 1.98 ± 0.73 new treatment/100,000 per year, respectively (Fig. 1).

### Comparison with paper notifications

The autonomous notifications collected before the informatization of RNAOC were entered into a dedicated electronic database and analyzed. Notifications referred to the period between 1983 and 2005, 4371 communications were about initiation of therapy, 6046 for follow-up visits. A total of 1266 communication of initiation of therapy with at least one follow-up visit were notified. Eight hundred and fifty communications (850) could not be entered because they lacked fundamental information as date of birth, initiation of treatment, etc. For what concerns the flux of information, there was an irregular distribution both in terms of notification/per year, with a peak of prevalence in 1991 (1.33/100,000), and of Regional contribution, with Sicily and Tuscany together providing about half of the notifications.

With regard to the information, only 727 of the notifications reported the diagnosis, with 43.7% of GHD and 32.7% Turner syndrome. Considering the scarce number of diagnosis, their distribution is not plausible and probably depends on a reduced number of communications about GHD.

The analysis of this database simply confirms that paper notifications are incomplete and incorrect, and an underestimation is evident.

## Discussion

Public health surveillance can be defined as the “… systematic, ongoing collection, management, analysis, and interpretation of data followed by the dissemination of these data to public health programs to stimulate public health action” [13]. This information is utilized for planning, implementing, and evaluating public health interventions and programs. Surveillance data are used both to determine the need for public health [14], for immediate public health action, for program planning and evaluation, and for formulating research hypotheses [15].

The collection of detailed clinical information is still heavily dependent on the surveillance tools used in the past years when paper, telephone and fax communication between public health staff and health care providers were the only ways to collect data. This approach is affected by a series of challenges, including the completeness and timeliness of reporting, the accuracy and specificity of data coding, the cost of data collection, and the incomplete reporting [16].

In recent years, technological and analytical innovations facilitated public health surveillance systems, especially requested in case of the need of rapid identification of communicable disease epidemics [17].

Our experience on the rGH prescriptions collected as paper notice confirms the challenges of the non-standardized communications in public health surveillance. In fact, when we entered the paper notifications in a dedicated electronic database to have a systematic collection, we observed that they were clearly incomplete in terms of both numbers and quality of information.

On these bases, a computerized registry on GH treatment requested by the Italian competent authority on medicines is crucial for pharmacosurveillance and requires setting up an online application, containing the key elements for rGH prescriptions as reported in the law. The advantages of an electronic entry and a central database dedicated to GH treatment rely on having a shared form, mitigating the probability of errors and allowing handling complete and correct data, meeting the need of combining technology, simplicity, privacy and data security. The quality control on data is facilitated by the controls still operating at RNAOC e-CRF level and subsequent check planned through specific cross-analysis between fields. In addition, this requirement is reducing in view of the updating of the system and the improvement of the users.

The RNAOC e-CRF has been adopted by almost all the Italian Regions, allowing collecting most of the notifications of GH therapy and implying a continuous interaction between a central institution, such as ISS, and the local authority. As a consequence, a constant updating about the authorization of the clinical centers to prescribe rGH therapy and their responsible is carried out. The number of users is wide and with different levels of activity and expertize in the RNAOC e-CRF and practical activities, accredited in Continuing Medical Education.

The national database is primarily an instrument of pharmacosurveillance, so that it was designed on the specific legal provision to provide policy- and decision-makers with complete and accurate data. Nevertheless, it can be also utilized for different purposes: (1) AIFA, which is the competent authority for decisions about medicines reimbursement and utilizations, receives, together with the conference of the regional council member for health, a detailed annual report on RNAOC data and activities, published by ISS; (2) regions are particularly interested in therapy appropriateness, which is directly related to the cost of rGH therapy, and could obtain statistical analysis to match or complete data from other public administration databases; (3) clinicians could visualize all the patients and follow-up visits related to their OU, in a clinical form.

We reported our preliminary clinical data on the essential information, such as treated subjects, visits, diagnosis and usage dose, to verify if the e-CRF RNAOC is reliably collecting rGH prescriptions.

At now, available data about prevalence of rGH treatment are not exhaustive and usually referred to specific range of age and diagnosis. The few data on incidence rates in GH deficiency in childhood are very scattered, varying from 1/2000–1/4000 to 1/30,000 people per year [18–21]. In Italy, Piedmont registry calculated a prevalence rate in patients under 18 for GHD of 9/10,000 and 2/10,000 of new diagnosis in 2002–2004 [22].

The prevalence and incidence rate of adult-onset GH deficiency is difficult to estimate. The addition of the prevalence data for pituitary macroadenoma (1:10,000 population) plus childhood-onset GH deficiency persisting into adult life gives an overall prevalence of 2–3/10,000 [23]. In a Danish nationwide study the incidence rates of childhood and adult onset GH deficiency was 2.15/100,000 and 1.65/100,000, respectively [24]. The Dutch National Registry of GH Treatment published data on adults, showing 200 subjects entering therapy every year with no calculation about prevalence [25]. Other data came from pharmaceutical company post-marketing registries and are focused essentially on efficacy and safety; thus, limited data are available on the epidemiology of GH treatment [1]. These disagreements depend on the differences in reference population, age and sex distribution, diagnosis, and starting age and duration of treatment, making the epidemiological data on rGH treatment between different databases difficult to compare.

Data from e-CRF RNAOC seem to fit into the range described in literature, making us confident in a nearly sufficient data collection, in terms of treated subjects, reported diagnoses and treatment.

Some problems are still present. One of the criticisms is that already active regional databases collecting rGH prescriptions are present in Piedmont, Lazio, Campania and Veneto. A transfer file has been designed to allow these regions to send the data useful for the national health surveillance system to the central database.

It is more than likely that RNAOC does not collect all the national prescriptions, partly because of the difficulties in including data from other databases and also for the lack of notifications due to overwork of the users having scarce time for entering data. Another reason could be a limited dissemination of information and knowledge about RNAOC.

Thus, further updating of the web system and more precise and automatic check and analysis are ongoing.

## Conclusions

The e-CRF of the web-based Italian Registry of rGH Treatment shows to be a functioning fundamental tool providing a exhaustive database of rGH treatment according to the law.

This national database represents a remarkable instrument for a correct and useful pharmacosurveillance, since data accuracy and completeness are crucial for ensuring both the correctness and epidemiological relevance of a given data set. This is a key point since knowledge about the number of subject in treatment and their diagnosis is mandatory for evaluating appropriateness, efficacy, and effectiveness of therapies.

In addition, a national database could be a powerful instrument to improve knowledge about patients with different diagnosis and therapeutic approaches, to explore new and useful aspects of this therapy, and to have an efficient survey on pharmacological abuses.
